# Association of early repolarization pattern and ventricular fibrillation in patients with vasospastic angina: A systematic review and meta‐analysis

**DOI:** 10.1002/clc.23804

**Published:** 2022-03-07

**Authors:** Guangqiang Wang, Na Zhao, Shu Zhong, Hua Wang

**Affiliations:** ^1^ Department of Cardiology The Affiliated Yantai Yuhuangding Hospital of Qingdao University Yantai China; ^2^ Department of Rheumatology The Affiliated Yantai Yuhuangding Hospital of Qingdao University Yantai China

**Keywords:** adverse cardiovascular events, early repolarization pattern, meta‐analysis, vasospastic angina, ventricular fibrillation

## Abstract

**Background:**

An early repolarization (ER) pattern is a risk factor for ventricular fibrillation (VF) in patients with vasospastic angina (VSA) caused by a coronary artery spasm. However, its detailed characteristics and prognostic value for VF remain unclear. Thus, we investigated the relationship between ER and VF in patients with VSA.

**Hypothesis:**

The ER pattern is associated with VF in patients with VSA.

**Methods:**

In this systematic review and meta‐analysis, we searched PubMed, Embase, Cochrane Library, and Web of Science databases for eligible studies published between January 2011 and December 2020; 8 studies with 1761 patients were included in the final analysis.

**Results:**

The ER pattern significantly predicted adverse cardiovascular events (ACEs) and VF (odds ratio [OR] = 5.13, 95% confidence interval [95% CI]: 3.16–8.35, *p* < .00001 and OR = 5.20, 95% CI: 3.05–8.87, *p* < .00001). The presence of ER in the inferior leads increased the VF risk (OR = 7.80, 95% CI: 4.04–15.05, *p* < .00001), regardless of the J‐point morphology or type of ST‐segment elevation in the ER pattern. A horizontal/descending ST‐segment elevation was significantly associated with VF in patients with or without an ER pattern during a coronary spasm (OR = 2.28, 95% CI: 1.07–4.88, *p* = .03). However, obstructive coronary artery disease was unrelated to the ER pattern (OR = 0.82, 95% CI: 0.27–2.53, *p* = .73).

**Conclusions:**

An ER pattern is significantly associated with an increased risk of ACE in patients with VSA. An inferior ER pattern with horizontal/descending ST‐segment elevation confers the highest risk for VF during VSA onset. Nevertheless, the ER pattern is not associated with obstructive coronary artery disease.

## 1 INTRODUCTION

An early repolarization (ER) pattern was previously regarded as a benign electrocardiogram (ECG) variant. It is characterized by J‐point elevation and QRS notching or slurring in the inferior and/or precordial leads, and it is often observed in teenagers and young adults (see Figure S1, Supplemental Content, which illustrates an ER pattern with J‐point and ST‐segment elevation).^1^, ^2^, ^3^ It is prevalent in 1%–13% of the general population, depending on age, race, and sex differences. Recently, the ER pattern in the inferior (Ⅱ, Ⅲ, and aVF) and/or lateral (Ⅰ, aVL, and V4‐6) leads has been determined to be an indicator of increased susceptibility to ventricular fibrillation (VF) during acute ischemia.^4^ Adverse cardiovascular events (ACEs), mainly including ventricular tachycardia (VT), VF, electrical storm, and sudden cardiac death (SCD), are associated with an increased risk of a poor cardiac prognosis. Previous studies have suggested that ACEs are attributed to an ischemic attack caused by a coronary artery spasm (CAS).^5^ Because of the fatal nature of ACEs, ECG markers that can distinguish between malignant forms of ER and acute ischemia are of prime clinical importance. Although this ECG pattern may not increase the risk for an immediate life‐threatening event, it may increase a patient's vulnerability to malignant arrhythmia during CAS. Vasospastic angina (VSA) is a unique form of acute transmural myocardial ischemia that is caused by CAS and it can occasionally trigger life‐threatening cardiac events.^5^ Although the incidence of life‐threatening arrhythmias is low (<10%), the frequency of the inferolateral ER pattern in VSA patients is 21.4%–26.0%. However, information on the prognostic significance of the ER pattern in patients with VSA is limited. Bastiaenen et al.^6^ declared that ECG territory, J‐wave amplitude, and ST‐segment morphology are associated with different degrees of risk for subsequent VF. Therefore, we performed an updated systematic review and meta‐analysis to assess whether the ER pattern and its detailed characteristics are associated with an increased risk of VF in patients with VSA.

## 2 METHODS

This meta‐analysis was conducted in accordance with the Preferred Reporting Items for Systematic Reviews and Meta‐Analyses statement.^7^ Ethical approval was not necessary, as this meta‐analysis used published data.

### 2.1 Eligibility criteria

Following the population/disease, intervention/exposure, comparison/control, outcome, and study design (PICOS) framework, studies were included if they: (1) were full‐text, English‐language studies; (2) were case–control or cohort studies (S); (3) reported patients with VSA diagnosed with VF (P) and showed comparisons between patients with ER pattern (I) versus those without (C); (4) reported the incidence of VF (O); (5) reported adjusted odds ratios (ORs), relative risks (RRs), or hazard ratios and their 95% confidence intervals (CIs) directly, or sufficient data that could indirectly calculate them. Meanwhile, studies were excluded if they were: (1) reviews, editorials, expert opinions, comments, case reports; (2) technical, cell, animal, or cadaver experiments; or (3) abstracts or conference proceedings.

### 2.2 Search strategy

The definition of ER pattern was redefined by Haïssaguerre et al.,^8^ to describe an association with idiopathic VF and the new definition has been almost exclusively used since then. Moreover, the ST‐segment and T‐wave morphology were reidentified by Tikkanen et al.^9^ We searched for articles published from January 2011 to December 2020, using the combination of subject headings and free words in the following databases: PubMed, EMBASE, Cochrane Library, and Web of Science. The search strategy used was (“variant angina” or “vasospastic angina” or “myocardial ischemia” or “Prinzmetal's angina”) AND (“ventricular fibrillation”) AND (“J‐waves” or “early repolarization”). Additional articles or abstracts were retrieved using hyperlinks and by manually scrutinizing the reference lists of relevant publications.

### 2.3 Study selection

Two investigators (N. Z. and S. Z.) systematically extracted the data in prespecified data forms. All potentially relevant reports were retrieved as complete manuscripts and, then, we assessed them according to the eligibility criteria. Any disagreements or uncertainties between the two investigators were resolved through consensus after rechecking the data source and consultation with a third investigator (G. W.).

### 2.4 Data extraction

Two investigators (N. Z. and S. Z.) independently extracted the related data using a predefined form. In each primary study, we extracted all the adjusted and unadjusted OR/RR and the corresponding 95% CI or data necessary to calculate it in this way (categorical), to evaluate ER in predicting the cardiac prognosis in the setting of VSA. The extracted data elements for each study included the first author's last name, publication year, the geographic location of study, study design, inclusion criteria, ER definition, exclusion criteria, total number of patients with VSA, patients' age and sex, high risk factors, left ventricular ejection fraction, medications, obstructive coronary artery disease, implantable cardioverter defibrillator, duration of follow‐up, adjusted variables, diagnosis of ER before or after VSA onset, endpoint events, and number of ACEs.

### 2.5 Quality assessment

We selected the Newcastle–Ottawa Scale (NOS), a nine‐score system, to assess the methodological quality of the included studies.^10^ Then, we evaluated the quality of the study in three domains: selection, comparability, and outcome or exposure. We assigned scores of 0–3, 4–6, and 7–9 for low, moderate, and high quality of studies, respectively.

### 2.6 Statistical analysis

All the potential references were imported into the EndNote software. We analyzed the data using Review Manager (RevMan version 5.3; Cochrane Collaboration) and adopted ORs and their 95% CIs as the effect quantities, which should be converted to their logarithms and SEs.^11^ The *I* ^2^ statistic and Cochran's *Q* test with its *p* were evaluated to determine statistical heterogeneity among studies.^12^ If the *I* ^2^ statistic and *p* were <50% and >.1, respectively, which means low heterogeneity existed, a fixed‐effects model was used in the meta‐analysis for pooling effect sizes. Otherwise, a random‐effects model was used. A funnel plot was used to assess the publication bias. Sensitivity analyses were conducted by omitting one study in turn to assess its effect on the overall results.^13^ When there was substantial heterogeneity, subgroup analyses were performed to seek the possible methodological and clinical heterogeneous estimates. A two‐sided *p* < .05 was considered significant. The Egger regression test was adopted to accurately judge potential publication bias using the Stata 15.0 software (StataCorp LP).^14^

## 3 RESULTS

### 3.1 Study selection and characteristics

The initial search yielded 118 potential studies. After screening the titles and abstracts, 18 studies were left for full‐text review. After reviewing the remaining 18 studies, 10 studies were excluded, because they were case reports or review articles and lacked available outcomes. Finally, a total of eight studies were included in the meta‐analysis.^15^, ^16^, ^17^, ^18^, ^19^, ^20^, ^21^, ^22^ The study selection process is shown in Figure S2 (see Figure and Supplemental Content, which illustrates the flow diagram of study selection).

All the selected studies were retrospective cohort studies, which were published between 2011 and 2020, with sample sizes ranging from 34 to 458 patients and a total of 1761 patients. All patients were from South Korea and Japan. Altogether, 966 patients who underwent coronary angiography with positive coronary spasm provocation tests were diagnosed with VSA. Among them, 251 patients with VSA showed an ER pattern in their ECGs. The main characteristics of the included studies are summarized in Table 1. We obtained original data by directly extracting from the included studies or by emailing the author. The detailed characteristics of the included studies are shown in Table S1 (see Table and Supplemental Content, which illustrates the characteristics of eight selected studies).

**Table 1:** Characteristics of the included studies in meta‐analysis

Author, year	Study design	Study location	Duration	Inclusion criteria	Definition of ER pattern	Exclusion criteria	Sample size (*n*)	VSA (*n*)	Diagnosis of ER pattern before or after VSA	ER pattern (*n*, %)	Follow‐up (months, mean ± SD)	Endpoint events	Covariate adjustment
Sato et al. (2011)^15^	Retrospective observational study	Japan	April 2007 to June 2010	Coronary spasm was defined as total or subtotal occlusion with delayed filling of the distal segment, and was associated with chest pain and/or ischemic ST‐segment elevation on ECG	J‐waves were defined as the positive deflection at the J‐point ≥0.1 mV above the isoelectric line in two or more contiguous leads, then they were classified as either the “notched” or “slurred.” The sites of J‐waves were indicated in the inferior leads (II, III, and aVF), right precordial leads (V1 and V2), left precordial leads (V3 –V6), and left lateral leads (I and aVL)	Bundle branch blocks, atrial fibrillation, Brugada syndrome, or Wolff –Parkinson–White syndrome	114	114	After	19 (16.7)	26 ± 10	Uneventful	Unadjusted
Oh et al. (2013)^16^	Retrospective observational study	South Korea	1995 to 2009	Coronary spasm is defined as transient, total, or subtotal occlusion (>90% stenosis) of a coronary artery with signs/symptoms of myocardial ischemia	ER patterns were stratified according to the degree of J‐point elevation (≥0.1 or >0.2 mV) that was either notched or slurred in at least two consecutive inferior or lateral leads. ST‐segment patterns after the J‐point were coded as follows: (1) horizontal/descending or (2) concave/rapidly ascending	Age > 70 years, Brugada syndrome, long and short QT syndrome, fixed coronary artery disease, and structural heart disease	281	281	Before	60 (21.35)	91.2 ± 56.4	Cardiac events	Age and sex
Inamura et al. (2015)^17^	Retrospective observational study	Japan	July 2010 to April 2014	Chest pain at rest and underwent coronary angiography with a positive provocation test for the diagnosis of VSA	ER was defined as a J‐point elevation ≥1 mm above baseline and slurring or notching of the terminal portion of QRS at ≥2 inferior (II, III, and aVF) and/or lateral (I, aVL, and V5–6) leads. The ST‐segment types were defined as “concave/rapidly ascending” and “horizontal/descending”	Bundle branch block, intraventricular conduction disturbances, or Wolff–Parkinson–White syndrome	116	66	After	31 (46.97)	26.1 ± 14	Arrhythmic events	Unadjusted
Kitamura et al. (2016)^18^	Retrospective observational survey	Japan	January 2003 to March 2014	VSA was defined as ≥90% narrowing of the epicardial coronary arteries on angiography performed during the provocation test for vasospasms, as well as the concomitant appearance of characteristic chest pain and/or ST‐segment deviation on ECG	An ER pattern was defined as an elevation of J‐point by ≥0.1 mV above the baseline, and/or either notching or slurring morphology of the terminal portion of QRS at ≥2 inferior (II, III, and aVF) and/or lateral (I, aVL, and V4 –V6) leads. The ST‐segment patterns were classified as “concave/rapidly ascending” and “horizontal/descending”	Hypertrophic cardiomyopathy	265	265	Before	64 (24.15)	66 ± 39.6	VF recurrences	Unadjusted
Fumimoto et al. (2017)^19^	Single‐center retrospective study	Japan	January 2002 to January 2014	A definite diagnosis was made based on the guidelines for VSA endorsed by the Japanese Circulation Society. Significant coronary stenosis was defined as a >50% luminal narrowing of the major coronary arteries evaluated by coronary angiography	J‐waves were defined as notching, slurring, or a J‐point elevation of 0.1 mV above the baseline in two contiguous inferior, lateral, or anterior leads	Atrial fibrillation, a paced rhythm, and intraventricular conduction block	166	62	Before	16 (25.81)	During provocation test	Arrhythmic events	Organic stenosis
Kamakura et al. (2018)^20^	Single‐center retrospective study	Japan	1996 to 2016	The diagnosis of CAS was based on a total or subtotal coronary artery narrowing (>90%) during the CAG, accompanied by ischemic electrocardiographic changes and/or chest pain, either spontaneously or in response to a provocative stimulus	The amplitude of inferolateral J wave or J‐point elevation had to be ≥1 mm or 0.1 mV above the baseline level, such as slurring or notching in any of the inferior (II, III, and aVF), lateral (V4, V5, and V6), and high lateral (I and aVL) leads. The QRS interval in patients with inferolateral J wave had to be <120 ms. A normal corrected QT interval was defined as ≥340 and <460 ms during sinus rhythm	Structural heart disease, Brugada syndrome, long/short‐QT syndrome, catecholaminergic polymorphic VT, commotio cordis, drug‐induced VF, or hypothermia; patients requiring catheter ablation because of frequent premature ventricular contractions (≥1000/day) originating from the Purkinje network or the ventricular outflow tract	34	34	After	21 (61.76)	92 ± 62	VF recurrences	Unadjusted
Shinohara et al. (2018)^21^	Single‐center retrospective observational study	Japan	January 2007 to December 2016	Variant angina was defined as patients who showed transient ST segment elevation on ECG during total or nearly total (99% stenosis with delay) occlusion by a coronary spasm provocation test according to Guidelines for the Diagnosis and Treatment of Patients with VSA set by the Japanese Circulation Society	The ER pattern was defined as “notching” or “slurring” with an amplitude ≥0.1 mV on the terminal QRS portion in ≥2 of the anterior leads (V1–3), inferior leads (II, III, and aVF), or lateral leads (I, aVL, and V4–6) when QRS duration is <120 ms. Patients having both notched type and slurred type J‐waves were classified as notched type	Brugada syndrome defined as coved‐type ST elevation in the anterior leads (V1–3)	458	50	Before	13 (26)	52 ± 33	Uneventful	Unadjusted
Ikeda et al. (2020)^22^	Single‐center retrospective observational study	Japan	November 2008 to April 2018	The definition of a positive diagnosis of coronary spastic angina was transient or complete or subtotal occlusion (>90% stenosis) with myocardial ischemia symptoms or ST changes in the ECG	ER was defined as an elevation of J‐point in at least two leads. The amplitude of the J‐point elevation of at least 1 mm (0.1 mV) above the baseline, was classified as slurring or notching in the inferior leads (II, III, and aVF) and lateral leads (I, aVL, and V4 to V6). The ST segments were defined as “upslope” and “horizontal/descending”	Complete right or left bundle branch block, Brugada syndrome, persistent atrial fibrillation, pacemaker implantation, Wolff–Perkinson–White syndrome, AMI, or lack of clinical data	327	94	Before	27 (28.72)	During provocation test	Arrhythmic events	Unadjusted

Abbreviations: AMI, acute myocardial infarction; CAS, coronary artery spasm; ECG, electrocardiogram; ER, early repolarization; VF, ventricular fibrillation; VSA, vasospastic angina; VT, ventricular tachycardia.

### 3.2 Methodological quality of included studies

The quality of the included studies, which were graded 6–9 scores in the NOS scale, is shown in Table S2 (see Table and Supplemental Content, which illustrates the methodological quality assessment of included studies by NOS). In addition, seven studies were considered of high quality and only one study with a score of 6 was considered of moderate quality.

### 3.3 Overall meta‐analysis

**Figure 1:**
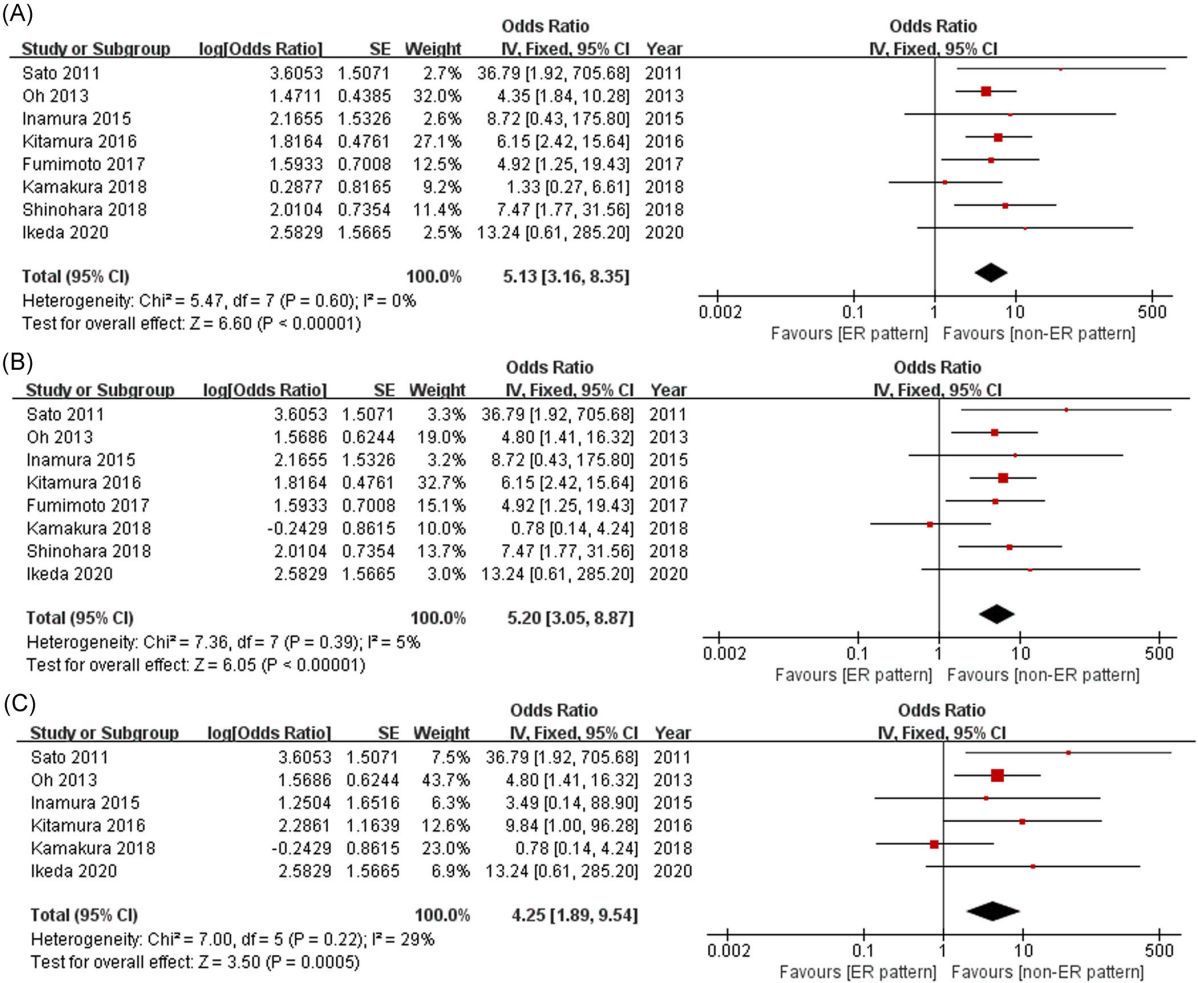
Forest plots demonstrating the associations between ER pattern and the occurrences of ACEs (A), VF (B), and follow‐up‐VF (C) in the patients with VSA. ACEs, adverse cardiovascular events; ER, early repolarization; VF, ventricular fibrillation; VSA, vasospastic angina

The overall meta‐analysis of the eight eligible studies was performed using a fixed‐effects model. Among the patients with VSA, the risk of ACEs was found to be nearly five times higher for those with an ER pattern as compared with those without an ER pattern (OR = 5.13, 95% CI: 3.16–8.35, *p* < .00001; Figure 1A). Moreover, the ER pattern was significantly associated with VF in patients with VSA (OR = 5.20, 95% CI: 3.05–8.87, *p* < .00001; Figure 1B). The ER pattern also showed a significant correlation with VF during the follow‐up (OR = 4.25, 95% CI: 1.89–9.54, *p* = .0005; Figure 1C).

### 3.4 Correlations between the ER pattern distribution and VF in patients with VSA

**Figure 2:**
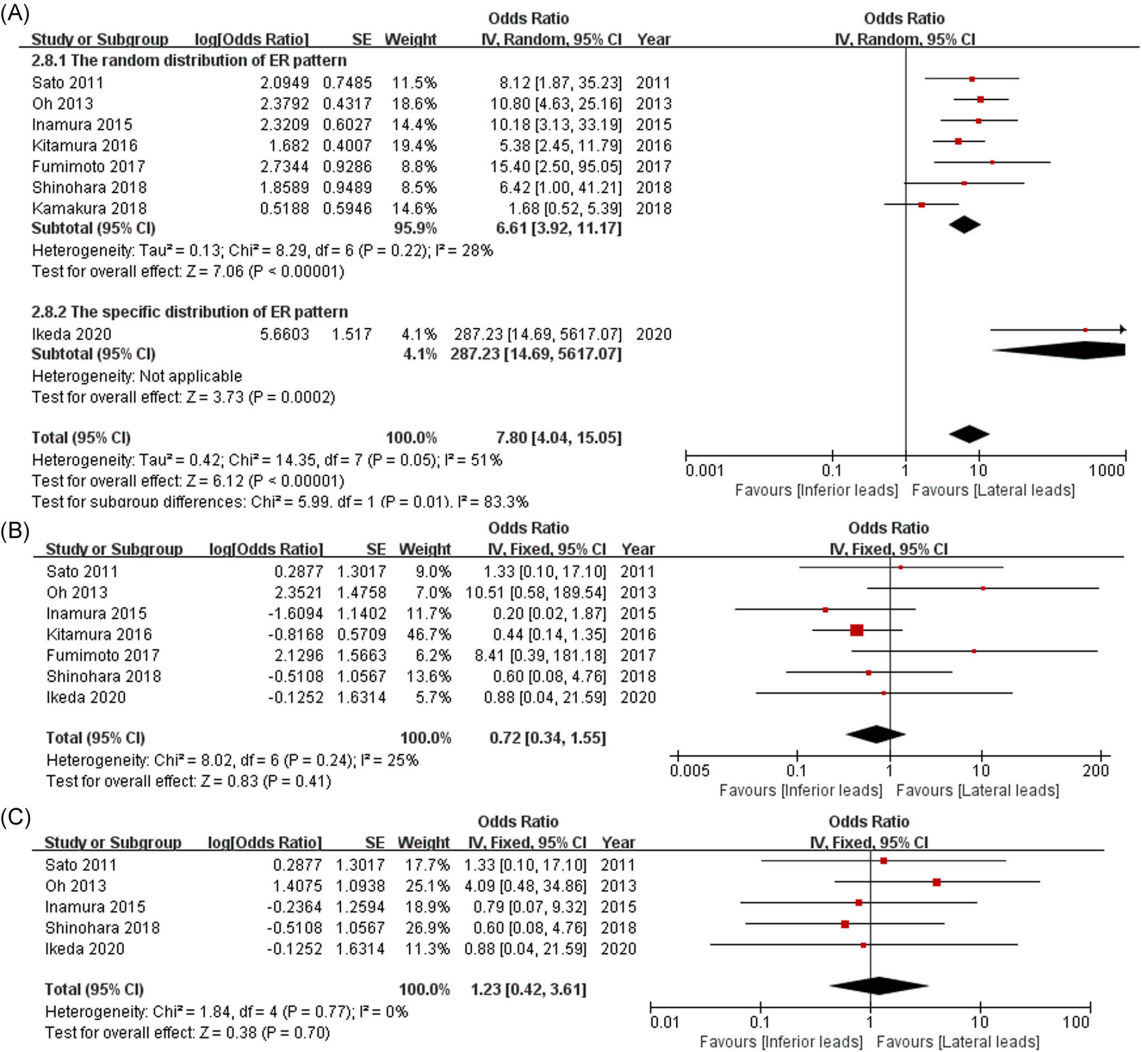
Forest plots comparing the impacts of two different ER locations (inferior vs. lateral leads) on the occurrence of VF in the setting of VSA (A), regardless of the morphology of J‐point (B) or type of ST‐segment elevation (C). ER, early repolarization; VF, ventricular fibrillation; VSA, vasospastic angina

Among the patients with VSA, the risk of VF was found to be nearly eight times higher in those with an ER pattern in the inferior leads as compared with those with an ER pattern in the lateral leads (Figure 2A; OR = 7.80, 95% CI: 4.04–15.05, *p* < .00001, random effect). However, the morphology of J‐point (OR = 0.72, 95% CI: 0.34–1.55, *p* = .41, fixed effect; Figure 2B) and the type of ST‐segment elevation (OR = 1.23, 95% CI: 0.42–3.61, *p* = .7, fixed effect; Figure 2C) for an inferior ER pattern were not associated with VF in patients with VSA. Thus, the location of the ER pattern was closely correlated with VF in patients with VSA.

### 3.5 Correlations between the morphology of J‐point and ST‐segment elevation, and VF in patients with an ER pattern during VSA onset

**Figure 3:**
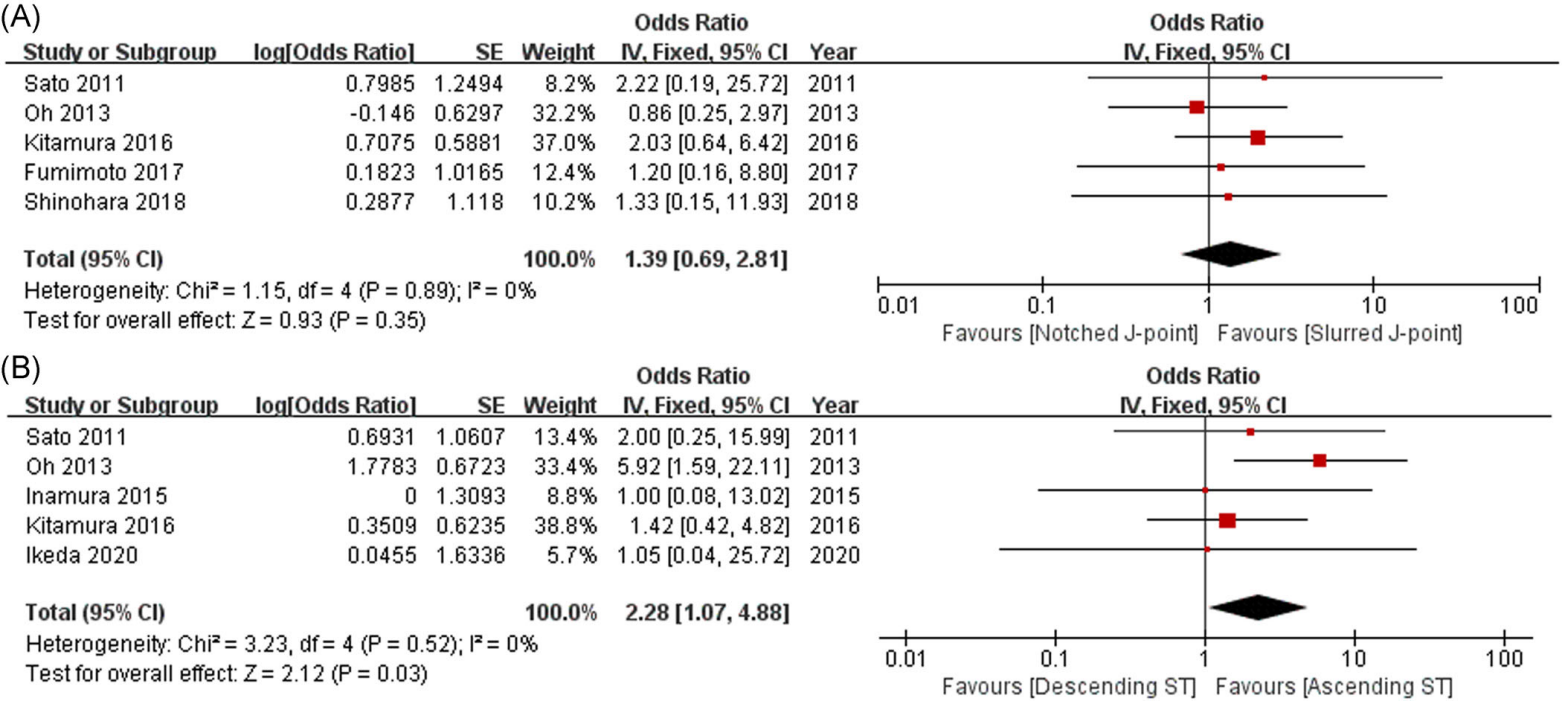
Forest plots showing the associations between the detailed characteristics of J‐point and ST‐segment elevation, and VF during VSA. (A) Morphology of J‐point; (B) type of ST‐segment elevation. ER, early repolarization; VF, ventricular fibrillation; VSA, vasospastic angina

The J‐point morphology of the ER pattern was not correlated with VF in patients with VSA (OR = 1.39, 95% CI: 0.69–2.81, *p* = .35; Figure 3A), although the horizontal/descending ST‐segment elevation was significantly correlated with VF during a coronary spasm (OR = 2.28, 95% CI: 1.07–4.88, *p* = .03; Figure 3B).

The amplitudes of the J‐wave (OR = 0.02, 95% CI: 0.02–0.06, *p* = .39) and of J‐waves ≥0.2 mV (OR = 1.86, 95% CI: 0.62–5.62, *p* = .27) were not correlated with VF in patients with an ER pattern with VSA (see Figure S3 and Supplemental Content, which illustrates the association between the amplitude of J‐wave and VF in ER patients with VSA). Thus, both studies revealed that J‐wave amplitude was not associated with VF in patients with an ER pattern during VSA onset. To date, there remains no cutoff amplitude for J‐waves that could accurately identify all high‐risk patients.^23^

### 3.6 Correlations of VF and ER pattern with obstructive coronary artery disease in patients with VSA

**Figure 4:**
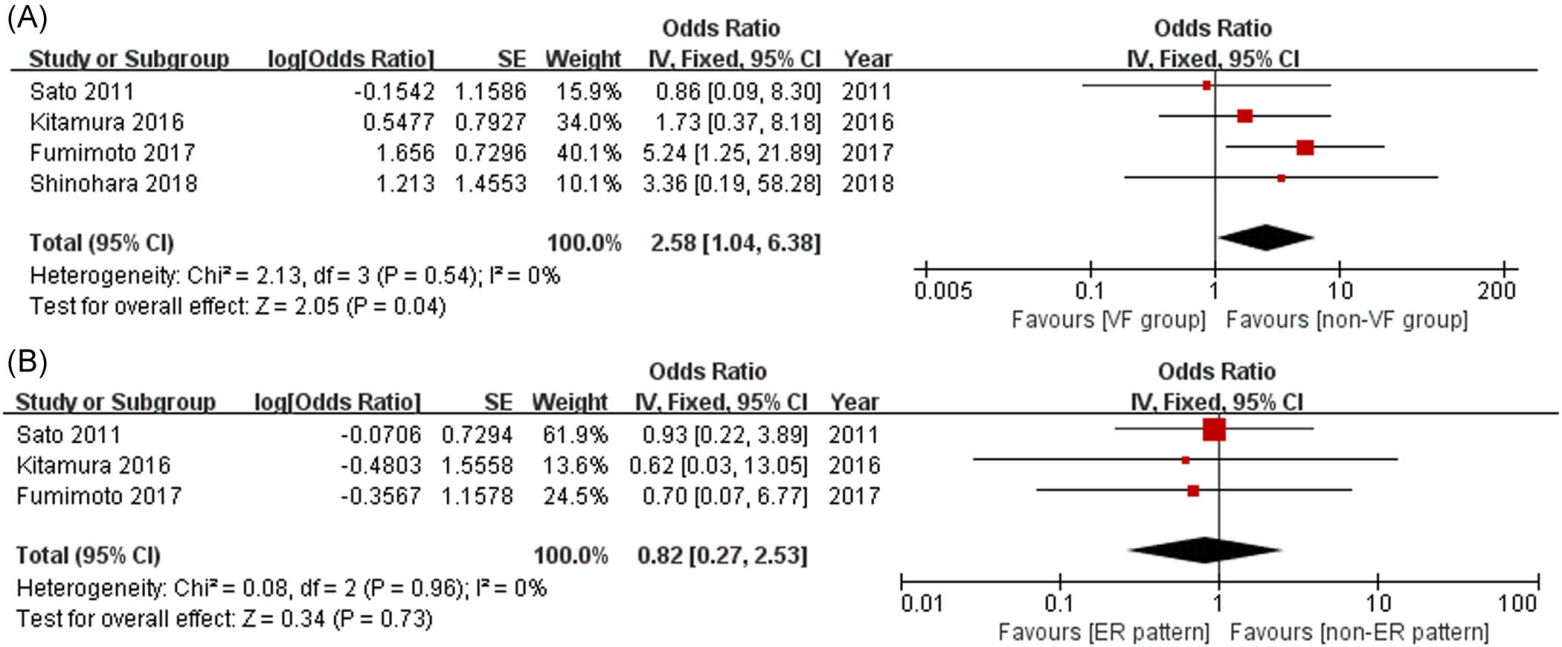
Forest plots revealing the correlations of obstructive coronary artery disease with VF (A) and ER pattern (B). ER, early repolarization; VF, ventricular fibrillation

VF was associated with obstructive coronary artery disease (OR = 2.58, 95% CI: 1.04–6.38, *p* = .04; Figure 4A). However, the ER pattern was not associated with obstructive coronary artery disease (OR = 0.82, 95% CI: 0.27–2.53, *p* = .73; Figure 4B).

### 3.7 Subgroup and sensitivity analyses

A subgroup analysis based on the distribution of the ER pattern was performed subsequently. Pooled analysis of the study by Ikeda et al.^22^ (OR = 287.23, 95% CI: 14.69–5,617.07, *p* = .0002; heterogeneity: *p* = .01, *I* ^2^ = 83.3%) revealed a 44‐fold higher risk of VF in patients with VSA with an ER pattern in the inferior leads as compared to in random patients with VSA (OR = 6.61, 95% CI: 3.92–11.17, *p* < .00001; heterogeneity: *p* = .22, *I* ^2^ = 28%; Figure 2A).

Sensitivity analysis was performed to assess the results of the meta‐analysis for the ACEs. After excluding each study, the results were consistent, thereby indicating reliability. Therefore, no significant heterogeneity was observed among the individual studies.

### 3.8 Assessment of publication bias

A funnel graph was plotted and the Egger's rank correlation test was performed to evaluate publication bias. For ACEs, the funnel plot was asymmetrical at its left middle and lower portions; this may be either due to publication bias or a true inexistence of negative studies. However, the Egger's test provided evidence of an absence of publication bias across the studies (*p* = .280; see Figure S4, Supplemental Content, which illustrates the visualized (A) and Egger's (B) funnel plots for publication bias).

## 4 DISCUSSION

### 4.1 Main findings

To the best of our knowledge, this is the first meta‐analysis to assess the relationship between ER pattern and VF in patients with VSA. All studies have consistently suggested that ER pattern was associated with ACEs. Especially, the inferior ER pattern was significantly associated with VF development. This meta‐analysis also confirmed that the ST‐segment morphology caused by CAS was associated with a high risk for subsequent VF.

### 4.2 Implications for clinical practice

Our meta‐analysis suggests that ER in the inferior leads correlate with significantly higher risk of VF during VSA compared with ER in lateral leads, although the individual lateral leads were slightly different in the included studies. The horizontal/descending ST‐segment elevation significantly increased the risk of VF in patients with or without an ER pattern during VSA onset. However, the J‐point morphology did not increase the risk of VF in ER patients. In addition, the J‐wave amplitude did not increase the risk of VF in ER patients during the onset of VSA, because the narrow time frame in which the dynamic ECG changes occur limits the ability to detect the ER pattern early.^24^ Thus, the typical J–ST–T pattern is characterized by J‐point elevation, a horizontal/descending ST‐segment configuration, and an inverted T‐wave during the onset of VSA. The morphology of the typical patterns (i.e., the “lambda‐like” and “triangular waveform” patterns) is varied (see Figure S5, Supplemental Content, which illustrates the schematic representations of the (A) lambda‐like and (B) triangular waveforms). Previous studies have shown that the above specific ECG patterns are associated with an increased risk of VF and they have been identified as predictors of a poor short‐term prognosis during acute myocardial infarction (AMI).^25^, ^26^, ^27^, ^28^, ^29^ The novelty of this meta‐analysis is that neither the J‐point morphology nor the J‐wave amplitude significantly increased the risk of VF in the ER patients with VSA. Moreover, the ER pattern is independent of obstructive coronary artery disease (angiographical stenosis > 50%).

In addition, our meta‐analysis demonstrated that VF is correlated with obstructive coronary artery disease. Thus, the triggering of VF by ER cannot be attributed to obstructive coronary artery disease in patients with VSA. Previous studies have revealed that the ER pattern that accompanies VSA is associated with VF or SCD, which provides potential substrates for malignant ventricular tachyarrhythmias with increased vulnerability and repolarization abnormalities.^17^ Conversely, Zhang et al.^30^ revealed that a notching ER pattern increased the risk of ventricular tachyarrhythmias in patients with AMI, and that it was mostly caused by obstructive coronary artery disease. The notching morphology of the J‐point may be related to obstructive coronary artery disease in ER patients with AMI. In addition, the progressive development of new pathologic Q and T wave changes is typically observed after AMI, although not after CAS.^31^

The results of this meta‐analysis also confirmed that the ST‐segment morphology had important value for ER pattern risk stratification during VSA onset.^32^ The ST‐segment elevation that is associated with a magnitude increase and R wave widening in the same lead, which resembles a monophasic curve at the peak of the attack, could represent a unique clue to the impending malignant arrhythmias.

### 4.3 Possible mechanisms

Vasospasm and ER pattern are two necessary conditions for the occurrence of VF, including acute ischemia and arrhythmogenic substrate. Recent evidence suggests that appearance of the ER pattern in patients with AMI indicates the presence of arrhythmic substrate, thus further increasing arrhythmic risk.^33^ In addition, ER pattern may aggravate the arrhythmogenic substrate in patients with VSA. Furthermore, the descending ST‐segment elevation may be an expression of an arrhythmogenic substrate in acute ischemia.^28^ Consequently, ER pattern may facilitate the identification of patients with VSA at a high risk for experiencing malignant arrhythmias.

### 4.4 Prospects for future study

The mechanisms underlying ER pattern development in acute ischemia remain unclear.^34^, ^35^ First, further experimental studies are needed to clarify the mechanism of different waveforms induced by CAS. Second, genetic analysis may provide deep insights into the underlying mechanisms of ER pattern. Moreover, genetic studies have focused upon candidate genes that might influence phase II of the action potential.^6^ The roles of candidate genes in ER pattern still need to be elucidated. Last, larger‐scale randomized controlled trials are required to prospectively validate the preliminary results of this meta‐analysis.

### 4.5 Study limitations

This study has several limitations. First, although we conducted a comprehensive literature search, the inevitable publication bias was difficult to rule out. Second, a subgroup analysis was performed by classifying the study by Ikeda et al.,^22^ where the specific patients with VSA with ER pattern in the inferior leads were selected. The analysis from the study by Ikeda et al.^22^ has showed that a significant influence on heterogeneity reduced from 51% to 28% across studies or in the overall results, and demonstrated that the included patients were a significant source of heterogeneity.

Third, most of the included studies did not provide adjusted data due to the high risk of confounding bias, such as age, sex, obstructive coronary artery disease, follow‐up time, and so on; thus, we did not use the adjusted data for further analysis. Fourth, we did not retrieve all databases due to permission restrictions, which might lead to the omission of other articles meeting eligibility criteria. Fifth, all of the included studies were conducted in Asian countries, but not in any Western country. A published review revealed that the patients with VSA were seen less frequently in the United States and Europe than in Japan.^5^ This finding suggested that Asian population might have a higher incidence of VSA than other populations. Last, there was a small sample size of included studies, which may affect the accuracy of our results to some extent.

## 5 CONCLUSION

ER pattern and ACEs in patients with VSA have a clear and strong association. An inferior ER pattern with horizontal/descending ST‐segment elevation confers the highest risk for VF during VSA onset. Nevertheless, ER pattern is not associated with obstructive coronary artery disease causing acute and persistent ischemia. Larger multicenter randomized clinical trials are required to elucidate the prognostic significance of ER pattern in patients with VSA.

## CONFLICT OF INTERESTS

The authors declare no conflict of interest.

## AUTHOR CONTRIBUTIONS

Guangqiang Wang, Na Zhao, Shu Zhong, and Hua Wang conceived of the design of the study. Guangqiang Wang and Na Zhao participated in the literature search and study selection. Na Zhao participated in data extraction and quality assessment. Guangqiang Wang and Na Zhao performed the statistical analysis. Guangqiang Wang finished the manuscript. All the authors read and approved the final manuscript.

## Supporting information

Supporting information.

Supporting information.

Supporting information.

Supporting information.

Supporting information.

Supporting information.

Supporting information.

Supporting information.

## Data Availability

The data supporting this meta‐analysis are from previously reported studies and data sets, which have been cited.
