# Supplementary figures and images for: Association of early repolarization pattern and ventricular fibrillation in patients with vasospastic angina: A systematic review and meta‐analysis
Source: Clin Cardiol. 2022 Mar 7;45(5):461–73. doi: 10.1002/clc.23804 (PMC9045077; doi:10.1002/clc.23804)

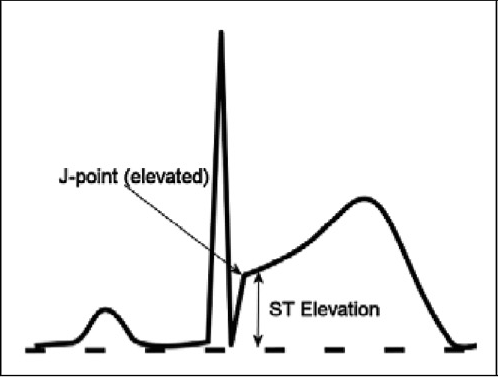

Supplement: Supplementary file 1 — Supporting information. [file CLC-45-461-s002.tif]

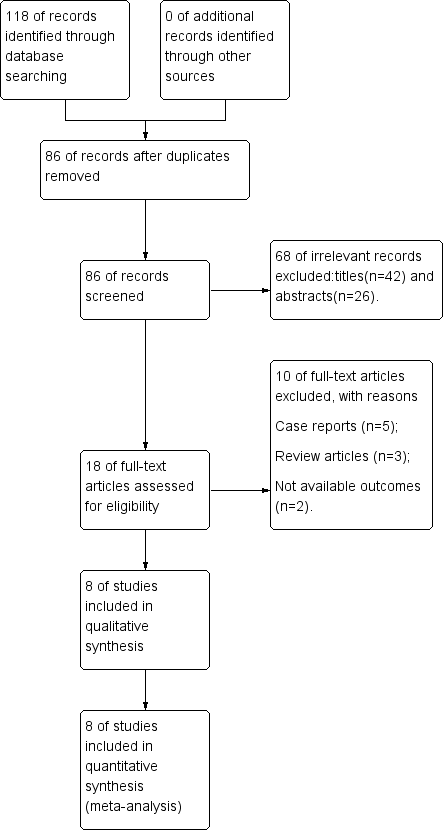

Supplement: Supplementary file 2 — Supporting information. [file CLC-45-461-s003.tif]

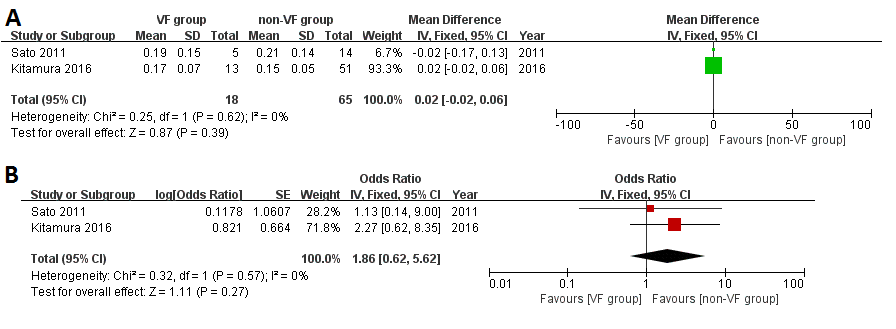

Supplement: Supplementary file 3 — Supporting information. [file CLC-45-461-s005.tif]

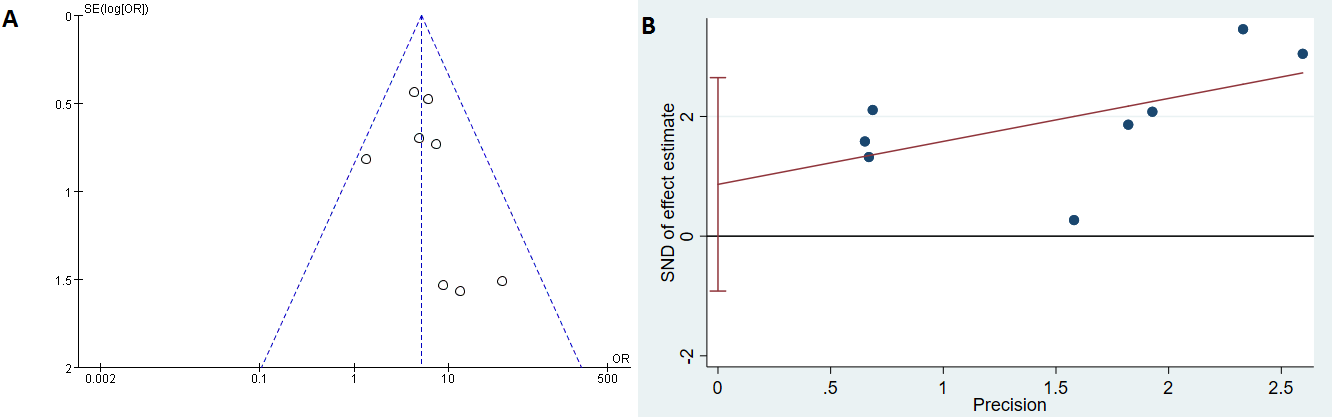

Supplement: Supplementary file 4 — Supporting information. [file CLC-45-461-s006.tif]

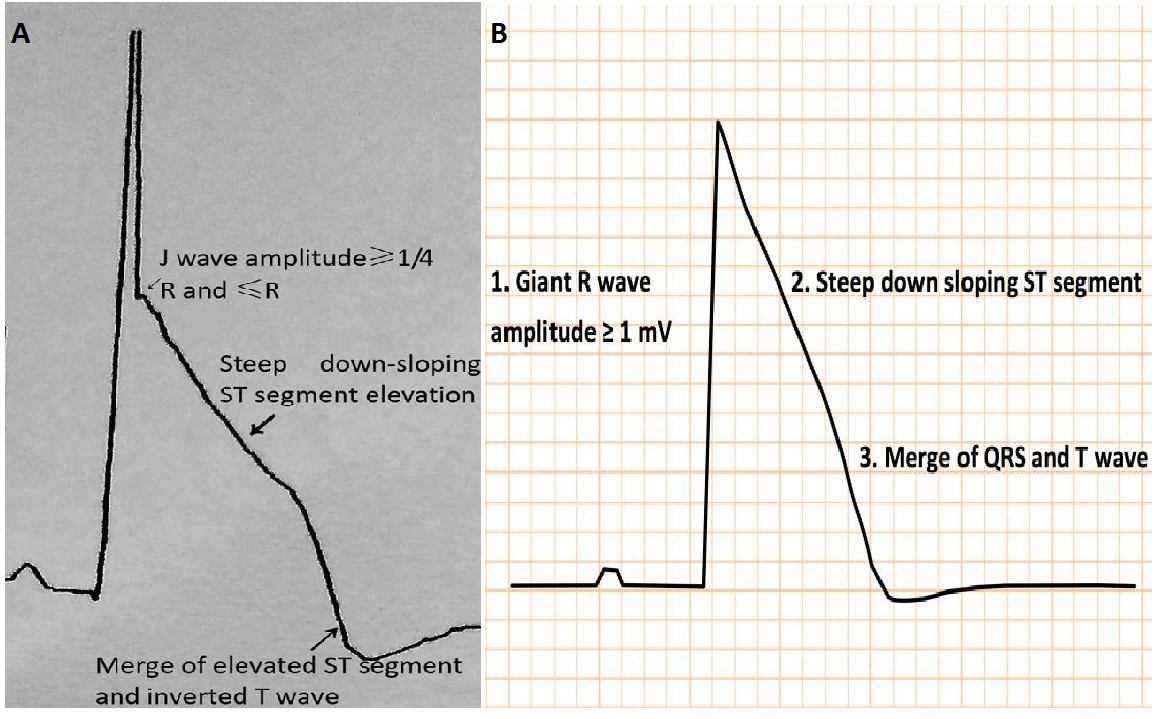

Supplement: Supplementary file 5 — Supporting information. [file CLC-45-461-s004.tif]
