# Supplementary material for: Association of early repolarization pattern and ventricular fibrillation in patients with vasospastic angina: A systematic review and meta‐analysis
Source: Clin Cardiol. 2022 Mar 7;45(5):461–73. doi: 10.1002/clc.23804 (PMC9045077; doi:10.1002/clc.23804)
Supplement: Supplementary file 6 — Supporting information. [file CLC-45-461-s007.docx]

|  |  |  |  |  |  |  |  |  |  |  |  |  |  |  |  |  |  |  |  |
| --- | --- | --- | --- | --- | --- | --- | --- | --- | --- | --- | --- | --- | --- | --- | --- | --- | --- | --- | --- |
|  | | | | | | | | | | | | | | | | | | | |
| Author,year | VSA(n) | Age of VSA patients(years,Mean±SD) | Age of ER patients(years,Mean±SD) | Male(n,%) | Family History of SCD(n) | Hypertension(n) | Diabetes(n) | Dyslipidemia(n) | Smoking(n) | Calcium channel blocker(n) | Nitrate(n) | LVEF(%) | Angiographical stenosis˃50%(n) | ICD(n) | Cardiac events(n) | Cardiac death(n) | VF(n) | Others(n) | VF currences during Follow-up(n) |
| Sato 2011 | 114 | NA | 60.9±7.6 | 52(45.6) | NA | NA | NA | NA | NA | 67 | 67 | NA | 15 | 0 | 5 | 0 | 5 | 0 | 5 |
| Oh 2013 | 281 | NA | 51.2±7.9 | 158(56.23) | NA | 124 | 30 | 111 | 91 | 275 | 252 | NA | NA | NA | 24 | 5 | 11 | 8 | 11 |
| Inamura 2015 | 66 | 63.9±11.4 | NA | 46(69.7) | NA | NA | NA | NA | NA | 66 | 66 | 64.9±9.9 | NA | NA | 3 | 0 | 3 | 0 | 1 |
| Kitamura 2016 | 265 | NA | NA | 173（65.28） | 19 | 138 | 28 | 119 | 160 | 215 | 76 | NA | 16 | 17 | 21 | 0 | 21 | 0 | 4 |
| Fumimoto 2017 | 62 | 59±13 | NA | 38(61.29) | 5 | 26 | 10 | 21 | 34 | 13 | 10 | 63±11 | 12 | NA | 11 | 0 | 11 | 0 | NA |
| Kamakura 2018 | 34 | NA | 42.7±17.4 | 30(88.24) | 3 | 9 | 3 | 8 | 20 | 13 | 13 | NA | 0 | 32 | 9 | 0 | 7 | 2 | 7 |
| Shinohara 2018 | 50 | NA | NA | 43（86） | 1 | 20 | 7 | NA | 30 | 50 | 50 | NA | 2 | 7 | 12 | 0 | 12 | 0 | 0 |
| Ikeda 2020 | 94 | 65.9±10.7 | NA | 5(56.4) | NA | 49 | 14 | 36 | 27 | 17 | 2 | 68.3±6.6 | NA | NA | 2 | 0 | 2 | 0 | 2 |
| VSA, vasospastic angina; ER, early repolarization; SD, standard deviation; SCD, sudden cardiac death; LVEF, left ventricular ejection fraction; ICD, implantable cardioverter defibrillator; VF, ventricular fibrillation; NA, not applicable. | | | | | | | | | | | | | | | | | | | |
|  |  |  |  |  |  |  |  |  |  |  |  |  |  |  |  |  |  |  |  |
