# Supplementary material for: Association of early repolarization pattern and ventricular fibrillation in patients with vasospastic angina: A systematic review and meta‐analysis
Source: Clin Cardiol. 2022 Mar 7;45(5):461–73. doi: 10.1002/clc.23804 (PMC9045077; doi:10.1002/clc.23804)
Supplement: Supplementary file 7 — Supporting information. [file CLC-45-461-s008.docx]

|  | | | | | | |  |  |  |
| --- | --- | --- | --- | --- | --- | --- | --- | --- | --- |
| Study | Selection | | | | Comparability | Outcome | | | Total score |
|  | Exposed cohort | Non-exposed cohort | Ascertainment of exposure | Outcome of interest |  | Assessment of outcome | Length of follow-up | Adequacy of follow-up |  |
| Sato 2011 | 1 | 1 | 1 | 1 | 1 | 1 | 1 | 1 | 8 |
| Oh 2013 | 1 | 1 | 1 | 1 | 1 | 1 | 1 | 1 | 8 |
| Inamura 2015 | 1 | 1 | 1 | 1 | 0 | 1 | 1 | 1 | 7 |
| Kitamura 2016 | 1 | 1 | 1 | 1 | 2 | 1 | 1 | 1 | 9 |
| Fumimoto 2017 | 1 | 1 | 1 | 1 | 2 | 1 | 0 | 0 | 7 |
| Kamakura 2018 | 1 | 1 | 1 | 1 | 2 | 1 | 1 | 1 | 9 |
| Shinohara 2018 | 1 | 1 | 1 | 1 | 1 | 1 | 1 | 1 | 8 |
| Ikeda 2020 | 1 | 1 | 1 | 1 | 1 | 1 | 0 | 0 | 6 |
|  |  |  |  |  |  |  |  |  |  |
