# Supplementary material for: Association of early repolarization pattern and ventricular fibrillation in patients with vasospastic angina: A systematic review and meta‐analysis
Source: Clin Cardiol. 2022 Mar 7;45(5):461–73. doi: 10.1002/clc.23804 (PMC9045077; doi:10.1002/clc.23804)
Supplement: Supplementary file 8 — Supporting information. [file CLC-45-461-s001.docx]

**FIGURE LEGEND**

**FIGURE S1** Early repolarization pattern with J-point and ST-segment elevation.

**FIGURE S2** Flow diagram of study selection.

**FIGURE S3**  Forest plots suggesting the association between the amplitude of J-wave and VF in patients with VSA with ER pattern. (A) Amplitude of J-wave; (B) amplitude of J-wave ≥ 0.2 mV. VF, ventricular fibrillation; VSA, vasospastic angina; ER, early repolarization.

**FIGURE S4**  Visualized (A) and Egger’s (B) funnel plots for publication bias.

**FIGURE S5** Schematic representations of the lambda-like (A) and triangular (B) waveforms.
